# Pediatric Average Volume Assured Pressure Support

**DOI:** 10.3389/fped.2022.868625

**Published:** 2022-05-04

**Authors:** Vishal Saddi, Ganesh Thambipillay, Bradley Martin, Gregory Blecher, Arthur Teng

**Affiliations:** ^1^Sydney Children's Hospital, Department of Sleep Medicine, Sydney, NSW, Australia; ^2^University of New South Wales, School of Women's and Children's Health, Kensington, NSW, Australia

**Keywords:** pediatrics, ventilation, sleep, AVAPS, infant, hypoventilation

## Abstract

Average volume assured pressure support (AVAPS) is a modality of non-invasive ventilation that enables the machine to deliver a pre-set tidal volume by adjusting the inspiratory pressure support within a set range. Data on its use in the pediatric population are limited to case reports and single centre case series. This article reviews paediatric data on use of AVAPS and highlights the need for validation to help develop specific guidelines on use of AVAPS in children.

## Introduction

The use of home non-invasive ventilation (NIV) has increased substantially in children over the last few decades, at least in part due to enhanced survival of children with chronic medical conditions along with improvements in home ventilator technology and provision of suitably sized pediatric masks (1, 2). When NIV is initiated, parameters are generally determined based on clinical assessment followed by an in-laboratory polysomnographic titration study where parameters are adjusted throughout the recording to determine optimal ventilatory settings for adequate gas exchange andupper airway patency (3). Although conventional fixed pressure NIV has been the mainstay therapy for children with neuromuscular and hypoventilation syndromes requiring respiratory support, several pediatric centres are reporting favorable outcomes with the use of average volume assured pressure support (AVAPS) for home ventilation (4–7). AVAPS enables the machine to deliver a pre-set tidal volume by automatically adjusting the inspiratory pressure support within a set range. It offers several advantages over fixed pressure NIV support such as its ability to compensate for the changes in tidal volume which occur with changes in lung compliance and sleep stages. It intuitively varies the inspiratory pressure, using higher pressure during REM sleep compared to NREM sleep resulting in a more stable ventilation and potentially improving adherence (5, 8). Despite its advantages, data on its use in children are sparse and recommendations on initiation and settings are extrapolated from adult experience.

## AVAPS Function and Settings

Rather than having a fixed IPAP setting, AVAPS has the capability to set a range of values for IPAP, a maximum and a minimum IPAP, to target delivery of a set tidal volume. Pressure support is no longer fixed and changes within the set parameters. The ventilator uses an inbuilt algorithm to either increase or decrease the inspiratory pressure from breath to breath to ensure delivery of the pre-set tidal volume^1^. The IPAP maximum also serves as a safety parameter to prevent barotrauma from excessive pressure. For the minimum IPAP delivery, the machine makes a selection from three pre-set algorithms (IPAP min; VT 60 ml/cmH_2_O + EPAP or 8 cmH_2_O + EPAP), choosing the highest value (9). Several additional parameters such as AVAPS rate of change ensure that patient-machine desynchronization is minimized and the IPAP pressure changes swiftly and efficiently to ensure patient comfort and optimal ventilation^1^. Tidal volume varies with each breath and it may take several breaths for AVAPS to attain the targeted tidal volume (10). EPAP is fixed similar to conventional BPAP, although there is an AVAPS auto-titrating EPAP (AVAPS AE) feature to regulate the EPAP as well in some devices (11).

The suggested settings are based on the information provided by the manufacturer and early adult studies^1^ (12, 13). In adults, a target tidal volume of 8 ml/kg of ideal body weight is recommended. The use of ideal body weight is to ensure optimal calculation of tidal volume for obese patients. The manufacturer provides pre-calculated ideal weights for a range of adult heights^1^. The maximum IPAP is 25 cmH_2_O and depending on the patient pathology can be set to a lower level. The minimum IPAP is set at EPAP +4 cmH_2_O, generally no <8 cmH_2_O. The set rate is generally 2 to 3 breaths below resting respiratory rate. The AVAPS rate of change determines the rate of change of IPAP and is kept shorter (range 1–5 cmH_2_O) for more unstable patients. The rise time is adjusted for patient comfort. The use of modem enabled devices help monitor pressures, air leaks and objective adherence to patient's healthcare provider. Recommendations for pediatric settings are lacking.

## Patient Selection

Although AVAPS debuted in 2003, a study published in 2006 by Storre et al. provided evidence of improvement in ventilation in a randomized crossover trial in adult patients with obesity hypoventilation syndrome. Adult studies have reported successful use in conditions such as chronic obstructive pulmonary disease (COPD), acute respiratory distress syndrome (ARDS), hypercapnia, encephalopathy, kyphoscoliosis and neuromuscular disorders (14–17). Pediatric studies have been limited to case series and case reports. A function similar to AVAPS, called iVAPS (intelligent volume assured pressure support), targets alveolar ventilation by adjusting pressure support (7). For purposes of this review, we have presented pediatric studies on volume assured pressure support ventilation below.

## Search Methods

A literature search of MEDLINE, Embase and PubMed was undertaken. No time limits were placed. The key terms included pediatrics, children, bilevel ventilation (BiPAP/BPAP), non-invasive ventilation and AVAPS. Inclusion criteria were: (1) AVAPS has been initiated either in an acute/subacute setting (pediatric intensive care unit) or electively (in stable setting, during or after a sleep study) and (2) English language articles and (3) Pediatric population (0–18 years). None of the studies were excluded from the review based on quality assessment. A total of 9 articles meeting the inclusion criteria were identified. These included 2 retrospective chart reviews, 1 prospective observational study and 6 case reports (Table 1).

**Table 1:** Literature review on pediatric use of AVAPS.

**Author study goal**	**Design**	**Study subjects**	**Measure of efficacy**	**Results**	**Conclusions and limitations**
Saddi et al. (4) Compare conventional BPAP with AVAPS	Retrospective case series	19 patients with difficult to control hypoventilation on conventional BPAP	Comparison of PSG parameters on BPAP and AVAPS. AVAPS was only used if hypoventilation was not well controlled on BPAP. Mean TcCO_2_ reduced from 55 mmHg in BPAP group. to 49 mmHg in AVAPS group.	AVAPS demonstrated better control of transcutaneous CO_2_ (TcCO_2_) compared to BPAP	AVAPS was better in controlling nocturnal hypoventilation. Limitations to the study include: single centre, retrospective design, lack of control group, the time period between comparison of two modes was long in some cases.
Sunkonkit et al. (5) Compare adherence between conventional BPAP with VAPS	Prospective observational study	20 children with neuromuscular disease	Comparison of PSG parameters and adherence data from machine download on BPAP and AVAPS. Adherence was recorded over a 3-month period. Adherence was 87% on BPAP compared to 100% on iVAPS for at least 4 h usage during a 3 month period.	Improved adherence on iVAPS compared to BPAP. No significant difference in gas exchange.	iVAPS associated with improved adherence. Limitations to the study include single centre, lack of control group, paediatric cohort only weighing more than 30 kg included in study due to device limitations and exclusion of one patient that could not tolerate iVAPS mode and another patient that was not adherent to NIV
Saddi et al. (23) AVAPS vs. BPAP in bronchopulmonary dysplasia (BPD)	Case report	Ex-24 week infant with severe BPD	Comparison of PSG recordings on BPAP and AVAPS. The comparison of TcCO_2_ recordings showed a more consistent ventilation on AVAPS.	AVAPS resulted in better TcCO_2_ compared to BPAP	AVAPS more efficient in controlling hypoventilation in young infants. Limitations include single centre and retrospective case study design.
Saddi et al. (8) AVAPS vs. BPAP in CCHS	Case report	10 month old infant with CCHS	Comparison of PSG recording on BPAP and AVAPS.	AVAPS associated with consistent TcCO_2_ reduction compared to BPAP	AVAPS may be a reliable alternative to conventional non-invasive BPAP in infants with CCHS. Limitations include single centre and retrospective case study design.
Stowe et al. (6) AVAPS vs. BPAP in ROHHAD	Case report	11 year-old girl with ROHHAD	PSG demonstrated significant hypoventilation with poor adherence to CPAP	AVAPS improved adherence, ventilation and pulmonary hypertension	AVAPS may improve ventilation and adherence compared to BPAP. Limitations include single centre and retrospective case study design.
Diaz-Abad et al. (24) AVAPS vs. CPAP in obese child with OSA	Case report	8 year-old obese child with OSA	PSG demonstrating hypoventilation not controlled on high CPAP pressures	AVAPS successful in treating OSA refractory to CPAP treatment	Use of AVAPS an alternative in cases of severe hypoventilation. Limitations include single centre and retrospective case study design.
Khayat et al. (7) iVAPS vs. BPAP in a cohort of patients with CCHS	Retrospective chart review	8 children with CCHS	Comparison of PSG recordings on BPAP and iVAPS. The TcCO_2_ recording showed a more consistent ventilation on AVAPS.	iVAPS associated with better reduction of TcCO_2_ compared to BPAP	iVAPS more efficient in reducing TcCO_2_ compared to BPAP. Limitations include inclusion of children weighing over 30 kg, lack of control group, single centre and retrospective study design.
Gentin et al. (25) AVAPS in congenital myopathy	Case report	3 year-old girl with multiminicore myopathy	PSG and machine download comparing gas exchange and pressure support on AVAPS and BPAP	AVAPS resulted in better TcCO_2_ compared to BPAP	AVAPS more efficient in managing hypoventilation. compared to BPAP. Limitations include single centre and retrospective case study design.
Vagiakis et al. (26) AVAPS in CCHS	Case Report	16-year-old girl with CCHS and tracheostomy	PSG recordings with capped tracheostomy on AVAPS.	Successful transition to non-invasive AVAPS from tracheostomy ventilation	AVAPS reliable mode of ventilation alternative to mechanical ventilation *via* tracheostomy in CCHS. Limitations include single centre and retrospective case study design.

## Device Considerations

The ability to reliably deliver small tidal volumes is critical to the use of AVAPS in pediatric populations. The minimum tidal volume threshold is 50 ml for the more advanced AVAPS-capable devices. Table 2 provides minimum tidal volume delivery for devices currently available with the AVAPS feature.

**Table 2:** Minimum tidal volume on AVAPS enabled devices.

**Device**	**Minimal tidal volume**
Trilogy Evo^®^	50 ml
Trilogy 100^®^	50 ml
BiPAP A40^®^	200 ml
BiPAP A40 Pro^®^	200 ml
Dreamstation BiPAP AVAPS^®^	200 ml

## Pediatric Settings

There are no pediatric specific guidelines for initiation of AVAPS. As discussed above, data on safety and reliability are based on single center studies with relatively small numbers. There are subtle differences between pediatric and adult AVAPS initiation that must be highlighted. The recommendation from the manufacturer to use ideal body weight (IBW) for calculation of tidal volume was presumably based on experience with obese adult patients. Although pediatric obesity is a growing problem, our centre has used AVAPS for a number of non-obese children. There is no universally accepted formula for calculation of IBW in pediatrics. A recent study published in JAMA Pediatrics comparing five different methods of calculating IBW in children revealed significant differences and variability in calculations using different methods (18). Furthermore, calculation of height is problematic for children with neuromuscular problems. Arm span is often used as a surrogate for height in children but upper limb contractures can make accurate measurements difficult. The data on arm span as a surrogate for height are based on small studies, mainly in children under 10 years of age (19, 20). One large study found ulnar length to be more reproducible and representative of height in healthy school age children, supporting its use in children with neuromuscular weakness (21). Furthermore, use of IBW for very young non-obese children may be unnecessary for calculation of tidal volumes. At our centre, we adopt the following approach:

1. For children with weight between 3rd and 95th centile we use actual body weight;
2. For children above healthy weight range (above 95th centile) or underweight (below 3rd centile) we use IBW for calculation of tidal volume as recommended;
3. For children shorter than 5 ft, the IBW in kg is calculated as ([height in centimeters]^2^ × 1.65)/1000. For boys taller than 5 ft, the IBW in kg is calculated as 39 + (2.27 × [height in inches – 60]). For girls taller than 5 ft, the IBW in kg is calculated as 42 + (2.27 × [height in inches – 60]) (22).

Other popular methods to calculate IBW include those proposed by McLaren, Moore, the American dietary association and the BMI 50 method (18). For children with disability where height cannot be measured, we use arm span as a surrogate for height measurement. It is important that height or arm span is measured meticulously to reduce errors in tidal volume calculation.

For the purposes of tidal volume calculation, a tidal volume of 6to 10 ml/kg is targeted (27). The IPAP minimum is calculated by adding 4 cmH_2_O to EPAP, similar to adults. The IPAP maximum depends on the patient's underlying condition with higher values for restrictive lung conditions.

In summary, the overall evidence on use of AVAPS in pediatric population remains confined to single centre and case studies without control groups. Prospective randomized control trials with long term follow up are needed. The recommendations and guidelines on initiation and titration of AVAPS in children are lacking. Further validations with data from other centres will help guide the optimal approach to use of AVAPS in pediatric settings.

## Author Contributions

VS drafted the initial manuscript. GT, BM, GB, and AT equally contributed to its development and approved the final version. All authors contributed to the article and approved the submitted version.

## Conflict of Interest

The authors have published several articles included in this review. The authors declare that the research was conducted in the absence of any commercial or financial relationships that could be construed as a potential conflict of interest.

## Publisher's Note

All claims expressed in this article are solely those of the authors and do not necessarily represent those of their affiliated organizations, or those of the publisher, the editors and the reviewers. Any product that may be evaluated in this article, or claim that may be made by its manufacturer, is not guaranteed or endorsed by the publisher.

## Footnotes

^1^ https://Philipsproductcontent.Blob.Core.Windows.net/Assets/20170523

## Abbreviations

CCHS: congenital central hypoventilation syndrome
ROHHAD: rapid-onset obesity with hypothalamic dysregulation, hypoventilation, and autonomic dysregulation.
